# Candidate gene analysis in a case of congenital absence of the endometrium

**DOI:** 10.1186/s40738-016-0015-8

**Published:** 2016-02-09

**Authors:** Serap Simavli, Ana Paula Abreu, Mary R. Kwaan, Robert G. Dluhy, Elena H. Yanushpolsky, Colleen Feltmate, Sandra R. Cerda, Rona S. Carroll, Ursula B. Kaiser, Wendy Kuohung

**Affiliations:** 1 Department of Obstetrics and Gynecology, Pamukkale University School of Medicine, Universty Street, Denizli, 20000 Turkey; 2 Division of Endocrinology, Diabetes and Hypertension, Brigham and Women’s Hospital and Harvard Medical School, Boston, MA 02115 USA; 3 Division of Colon and Rectal Surgery, Department of Surgery, University of Minnesota, Minneapolis, MN USA; 4 Department of Obstetrics and Gynecology, Brigham and Women’s Hospital, Boston, MA 02115 USA; 5 Department of Pathology and Laboratory Medicine, Boston University School of Medicine, Boston, MA USA; 6 Department of Obstetrics and Gynecology, Boston University School of Medicine, 85 E. Concord St, 6th floor, Boston, MA 02118 USA

**Keywords:** Absence of endometrium, Primary amenorrhea, Congenital uterine anomaly

## Abstract

**Background:**

Primary amenorrhea usually result from a genetic or anatomic abnormality. We present the first reported patient with the absence of endometrium and lumen in a small bicornuate uterus in a patient with primary amenorrhea.

**Case presentation:**

A 41-year-old woman presented for evaluation of primary amenorrhea and infertility. She did develop normal secondary sexual characteristics but never had menses. Physical examination, hormone analyses, and karyotype analysis were normal. Transvaginal ultrasonography revealed a small uterus with absent endometrial stripe. Ovaries were normal in size. Pathology from hysterectomy for abnormal Pap smears revealed a hypoplastic bicornuate uterus with absence of lumen and absent endometrium. DNA analyses for mutations in the coding sequences of three members of HOXA gene family was performed, but no variants in the coding sequence of these genes were found. These findings support the hypothesis that mutations in the coding sequence of HOXA10, HOXA11, and HOXA13 are not responsible for congenital endometrial absence with bicornuate hypoplastic uterus.

**Conclusions:**

Congenital absence of the endometrium is an uncommon etiology for primary amenorrhea, and nonvisualization of the endometrial stripe on ultrasound imaging in association with primary amenorrhea should raise suspicion of this rare disorder in this case.

## Background

Development of phenotypic sex in humans is tightly controlled by genetic factors. Normal development of the female reproductive tract involves a series of complex interactions that direct differentiation of the mullerian ducts and urogenital sinus (UGS) to form the internal female reproductive tract. The mullerian ducts differentiate to form the fallopian tubes, uterus, uterine cervix, and superior aspect of the vagina, while the UGS differentiates to form the mid and inferior vagina. When dysregulation or interruption occurs in any of the dynamic processes of differentiation, migration, fusion, or canalization, a wide spectrum of mullerian duct anomalies can result. Mayer-Rokitansky-Kuster-Hauser syndrome (MRKH syndrome) is characterized by mullerian duct agenesis. External genitalia and secondary sexual characteristics are normal in most cases. Primary amenorrhea in an adolescent girl is the most usual presentation. The incidence of MRKH syndrome is 1 in 5000 female live births [1].

Despite the existence of many hypotheses and candidate genes, the etiology of most cases of MRKH syndrome remains unknown. Although most cases of MRKH syndrome are sporadic, some familial cases have been described, indicating a possible genetic cause. Several genes have been investigated for mutations in MRKH syndrome, including AMH, AMHR, CTRF, WT1, PBX1, GALT, RAR-gamma, RXR-alpha, the WNT gene family, and the homeobox (HOX) gene family. Only the WNT and HOX genes have been clearly implicated in MRKH syndrome [2–5]. Although Wnt4, Wnt5a, Wnt7a, and Wnt9b have been demonstrated to be required in the development of female reproductive organs in mice, in humans mutations in only the WNT4 gene have been shown to be associated with cases of MRKH syndrome [2, 6]. The phenotypic presentation of the WNT4 mutation included mullerian duct hypoplasia or agenesis associated with clinical and/or biologic evidence of hyperandrogenism [2, 6]. WNT4 mutations were not found in MRKH patients without hyperandrogenism [7, 8]. Because our patient had no clinical and/or biologic evidence of hyperandrogenism, we aimed to search for mutations in the *HOXA* gene family (HOXA10, HOXA11, and HOXA13). In the present case, we report the absence of endometrium and lumen in a small complete bicornuate uterus in the spectrum of presentation of MRKH and non communicating uterine horns, in a patient with primary amenorrhea. This anomaly results from only partial fusion of the mullerian ducts. This leads to a variable degree of separation of the uterine horns that can be complete as current case [9]. Pregnancy outcomes have been reported similar with the general population. But, some women do develop complications, such as pregnancy loss, preterm labor, or malpresentations [10]. To the best of our knowledge, endometrial absence in women with abnormal uteri has not been reported previously. We made cytogenetic analysis of *HOXA* genes as candidate genes involved in the development of this unusual mullerian anomaly.

## Case presentation

A 41 year old woman of mixed Asian/Russian descent presented for evaluation for primary amenorrhea. She reported normal development of pubic and axillary hair and normal breast development, but she never had menses. She reported that she underwent evaluation for primary amenorrhea when she was 18 years old in Russia. She was found to have an extremely small uterus with normal hormone levels, and she did not have withdrawal bleeding after a course of progestin. Primary amenorrhea was thought likely to be related to her uterine abnormality or possibly related to hypothalamic dysfunction. She denied a history of diethylstilbestrol (DES) exposure. She had a history of genital herpes and abnormal Pap smears showing a high grade squamous intraepithelial lesion and positive for HPV-16 and−18. Her medical history was significant for low bone mass, diagnosed at age 39 years. Family history was noncontributory.

The patient’s height was 157.5 cm inches and she weighed 41.7 kg Her body mass index was 16.9 kg/m,^2^considered underweight. Her physical examination was normal, with normal sexual hair development and Tanner stage five breasts. There were no skeletal deformities. Gynecologic examination showed a normal vulva and vagina and a small cervix with a visible central os.

Hormonal testing was normal, with follicle-stimulating hormone (FSH) 3.3 mIU/mL (normal range 3.9–13.2 mIU/mL), luteinizing hormone (LH) 3.4 mIU/mL (2.4–12.6 mIU/mL), estradiol 101 pg/mL (14–188 pg/mL), thyroid stimulating hormone (TSH) 1.12 mIU/mL (0.5–6 uU/ml), prolactin 11 ng/mL (3.3–26.7 ng/mL), and 25-hydroxy-vitamin D 25 ng/ml. (25–80 ng/mL). She did not have withdrawal bleeding after a course of progestin. Karyotype was normal female 46,XX. Bone mineral density as measured by DEXA revealed a femoral neck T-score of−2.2 and total hip of−2.2. The low T-scores were thought to not necessarily reflect decreased bone strength due to her small frame and Asian background. Transvaginal ultrasonography demonstrated a hypoplastic uterus measuring 2.2 x 2.1 x 0.7 cm with no visible endometrial stripe and bilateral normal sized ovaries (left ovary measuring 3.1 x 2.1 x 1.8 cm, right ovary measuring 4.1 x2 .2 x 1.8 cm) with follicular echo. Abdominopelvic urogram demonsrated no renal or other organ abnormality.

She was seen by a reproductive endocrinologist for a pre-operative evaluation. Her colposcopy proved to be difficult because the cervix was flush with the vaginal vault. Cervical biopsy pathology was consistent with a high grade squamous intraepithelial lesion (CIN II-III), and the decision was made to proceed to hysterectomy for persistently abnormal Pap smears. The patient underwent abdominal hysterectomy. Findings at surgery included normal appearing fallopian tubes and ovaries, so these were not removed at surgery. The uterus appeared small and bicornuate. Her postoperative course was uneventful, and she was discharged home on the 4^th^ postoperative day.

**Fig. 1:**
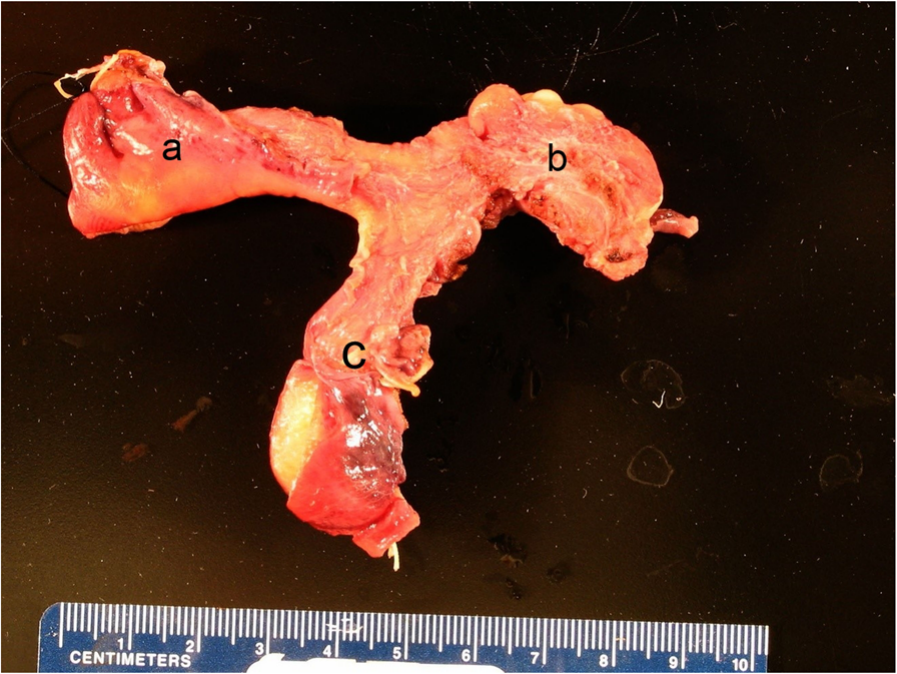
Gross image of the patient’s bicornuate uterus without endometrium or endometrial cavity; **a** right uterine cornu, **b** left uterine cornu, **c** cervix

**Fig. 2:**
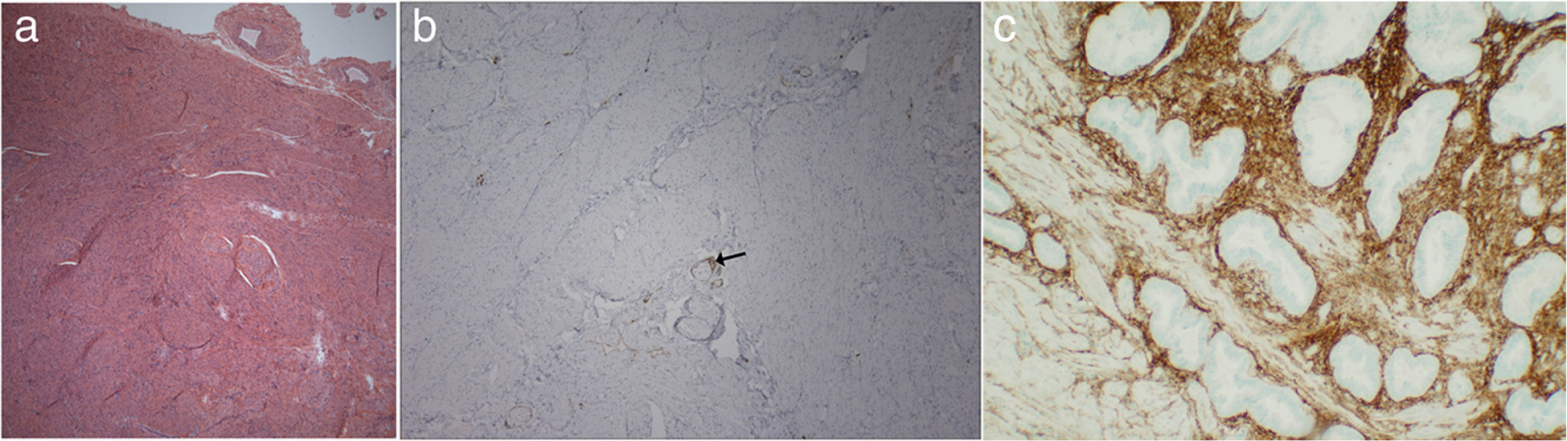
Uterine histopathology. **a** Myometrial tissue with absence of endometrium (hematoxylin and eosin stain, magnification 4x). **b** Absence of staining for CD10, a marker for endometrial stroma. CD10 staining is present only around vessels (*arrow*), and there is no evidence of endometrium (magnification 10x). **c** Normal uterine section showing staining for CD10 in endometrial stroma for comparison. (magnification 20x)

Pathology was reported as uterus bicornis unicollis with no endometrial tissue or lumen identified neither the fused portion of uterus, nor separate cornua of the uterus with immature endocervical epithelium in the cervix (Fig. 1). In the vaginal apex, vaginal intraepithelial neoplasia (VAIN) III was identified that extended to the vaginal margin. On deeper tissue sections, no endometrium nor lumen were identified in fused portion and separate cornua of the uterus (Figs. 2a and b). A section of normal endometrial stroma staining with CD10 is included for comparison (Fig. 2c).

### Genetic testing

Partners Human Research Committee approval was obtained for blood drawing and genetic and molecular investigations. Written informed consent was obtained, and blood was drawn from the patient. DNA was extracted, and lymphocytes were transformed to create an immortalized cell line using standard techniques [11]. Polymerase chain reaction (PCR) was performed using published primers [12] to amplify the entire coding region of HOX genes involved in the development of the mammalian female reproductive system (HOXA10, HOXA11, HOXA13). Products were sent for sequencing to search for base-pair changes. We did not identify any variants, including synonymous mutations, nonsynonymous mutations, deletions, insertions, or single-nucleotide polymorphisms, in the coding sequences of these genes.

## Conclusions

We present the first reported patient with primary amenorrhea due to complete absence of endometrium and lumen in a small bicornuate uterus. Congenital absence of the endometrium is a rare cause of primary amenorrhea. Berker et al. [13] reported a case with normal uterine size and shape with a sclerotic uterine cavity lacking endometrium. Our patient differed from this case in that she had a small bicornuate uterus without a uterine cavity.

Primary amenorrhea usually results from a genetic or anatomic abnormality. Causes of primary amenorrhea include hypergonadotropic hypogonadism, hypogonadotropic hypogonadism, and eugonadism with an anatomic abnormality. Female reproductive tract abnormalities account for about one-fifth of primary amenorrhea cases. MRKH syndrome, the second most common cause of primary amenorrhea, is characterized by the agenesis or partial agenesis of the mullerian duct system. Patients with MRKH syndrome present with normal development of secondary sexual characteristics and a normal 46,XX karyotype, similar to the present case [14, 15].

The HOX gene family is expressed in the mouse and human adult reproductive tract with a similar expression pattern as in the embryo. HOXA9 is expressed in the fallopian tubes, HOXA10 in the developing uterus, HOXA11 in the lower uterine segment and cervix, and HOXA13 in the ectocervix and upper vagina [16]. Abnormalities in the development of mullerian structures were observed in mice with Hox gene mutations [17]. Hoxa11 mutant female mice display normal uteri at birth, but decreased cellular proliferation/increased apoptosis result in the absence of stromal tissue by day 21 after birth [18]. Hoxa11 mutant mice were sterile, likely due to reduced stromal tissue and endometrial gland development in the uteri [19, 20].

In humans, HOXA10 and HOXA11 have been shown to be necessary for normal development of the endometrium [21], while DNA sequence variations in HOXA13 were identified in patients with hand–foot–genital syndrome and mullerian duct fusion defects [22]. Cheng et al. identified a gene mutation in the coding region of the HOXA10 gene in a Chinese woman with uterine didelphys; this mutation had a documented frequency of one out of 109 Chinese patients [23]. They showed that this mutation diminished the transactivation activity of HOXA10 in a luciferase assay. Likewise, Ekici et al. [4] sequenced the HOXA10 and HOXA13 genes of patients with MRKH syndrome and non-MRKH patients with genital malformations; HOXA10 and HOXA13 DNA sequence variations were found in patients with genital malformations. This suggests that rare DNA sequence variations in the HOXA10 gene may contribute to abnormal development of the female internal genitalia. In another study, Liatsikos et al. [24] analyzed the HOXA10 and HOXA11 genes in patients with various genital anomalies and found a heterozygous HOXA11 gene mutation resulting in a non-synonymous amino acid substitution (p.Pro38Arg) in a patient with a septate uterus. Although rare, heterozygous mutations of the HOXA10, HOXA11 and HOXA13 genes have been associated with genital malformations [22–26]; however, we did not find any DNA sequence variations in the present case.

We investigated only HOXA genes in this study. Other genes such as WNT5a and WNT7a that have been implicated in the glandular development of the uterus and that have a critical role in guiding epithelial-mesenchymal interactions [27, 28] may be involved in the genesis of this anomaly. Non-genetic factors such as perinatal exposure to toxins may also have given rise to this anomaly.

Here we report, to our knowledge, the first patient with congenital endometrial absence with hypoplastic uterus in the literature which may be the variant of MRKH without functional endometrium. The complete absence of endometrium in the uterus is a rare cause of primary amenorrhea and infertility. Absence of endometrium should be considered in women with primary amenorrhea in the presence of a uterus and cervix that cannot be explained by any of the known hormonal or genetic etiologies and who fail to respond to estrogen/progesterone withdrawal challenge.

### Ethics approval and consent to participate

Partners Human Research Committee approval was obtained for blood drawing and genetic and molecular investigations. Written informed consent was obtained from the patient.

## Abbreviations

DES: Diethylstilbestrol
DEXA: Dual-energy X-ray absorptiometry.
FSH: Follicle stimulating hormone
LH: Luteinizing hormone
MRKH syndrome: Mayer-Rokitansky-Kuster-Hauser syndrome
TSH: Thyroid stimulating hormone
UGS: Urogenital sinüs
